# Antifungal activity of TiO_2_/AgBr nanocomposites on some phytopathogenic fungi

**DOI:** 10.1002/fsn3.2357

**Published:** 2021-06-01

**Authors:** Aziz Habibi‐Yangjeh, Mahdi Davari, Reza Manafi‐Yeldagermani, Shervin Alikhah Asl, Samira Enaiati, Asgar Ebadollahi, Solmaz Feizpoor

**Affiliations:** ^1^ Department of Chemistry Faculty of Science University of Mohaghegh Ardabili Ardabil Iran; ^2^ Department of Plant Protection Faculty of Agriculture and Natural Resources University of Mohaghegh Ardabili Ardabil Iran; ^3^ Department of Plant Sciences Moghan College of Agriculture and Natural Resources University of Mohaghegh Ardabili Ardabil Iran

**Keywords:** agrochemicals, antifungal activity, crop protection, fungicides, *Fusarium graminearum*, TiO_2_/AgBr

## Abstract

TiO_2_/AgBr composites were synthesized by a simple ultrasonic strategy. Various instruments such as SEM, EDX, XRD, and FT‐IR were exploited to investigate their characteristics. Antifungal activities of the as‐obtained samples were assessed through the inactivation of *Fusarium graminearum* in the spore suspension method and mycelial growth inhibition of *F. graminearum*, *Botrytis cinerea*, and *Sclerotinia sclerotiorum* in the microdilution method. The results represented that the TiO_2_/AgBr samples possess higher antifungal activities on *F. graminearum* spores than the pure TiO_2_. The sample with 20 wt% silver bromide represented the highest inhibitory effect on the growth of *F. graminearum* so that all fungal spores were degraded in the initial times of the treatment process. The inactivation of fungal spores after 60 min was 35.2%, 97.8%, 98.9%, and 98.7%, in respect, for 5, 10, 20, and 30 weight percent of AgBr in the binary nanocomposites, while the inhibition rate was 13.4% for the pure TiO2. With increasing ultrasound irradiation time for more than 30 min, the inactivation rate constant decreased. It was also found that the antifungal activity of the nanocomposites without calcination was higher than those of the calcined materials. Considering the antifungal potential against phytopathogenic fungi and advantages such as simple synthesis and eco‐friendly nature, it seems that TiO_2_/AgBr nanocomposites can be used instead of synthetic chemicals after additional field investigations and mass production.

## Graphical abstract

TiO_2_/AgBr nanocomposites synthesized by simple ultrasonic strategy showed the high antifungal activity against some phytopathogenic fungi and the sample with 20 wt% of AgBr represented the highest inhibitory effect.

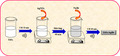

## 1 INTRODUCTION

The diminishment of agricultural products caused by different phytopathogenic agents remains a severe challenge in recent years. Among the pathogens, fungi have the most significant role in crop production losses (Bebber & Gurr, 2015). *Fusarium graminearum*, as a causal agent of Fusarium head blight, has become a major limiting factor for sustainable wheat production around the world (Davari et al., 2013). *Sclerotinia sclerotiorum* and *Botrytis cinerea* are common necrotrophic fungal plant pathogens that can attack a wide range of plant species during crop cultivation and harvested productions (Amselem et al., 2011). Although synthetic fungicides are widely utilized for fungal plant diseases management, some disadvantages such as harmful effects on the environment and human health, high production costs, and appearance of resistant strains have attracted researchers to design more efficient strategies (Bartlett et al., 2002; Joo, 2005; Le & Bach, 2019; Sharma et al., 2009).

Nowadays, the use of nanomaterials as efficient and eco‐friendly agents in the control of plant pathogens has been successfully studied (Balaure et al., 2017; Hayles et al., 2017; Khan & Rizvi, 2014; Servin et al., 2015; Singh et al., 2015; Sinha et al., 2017; Worrall et al., 2018). Among the nanomaterials, titanium dioxide (TiO_2_) has been gained great attention compared to CuO, ZnO, etc. to reduce the fungal diseases, because of its low cost, eco‐friendly nature, unique physicochemical properties, and high stability (Darbari et al., 2011; Huang et al., 2013). Despite the appealing features, TiO_2_ revealed low antifungal efficiency in practical applications (Beltrán‐Partida et al., 2017). To overcome this problem and boost the antifungal activity, the integration of TiO_2_ with suitable nanomaterials has been suggested. Silver‐based semiconductors are an excellent choice to increase the antifungal activity of TiO_2_ due to their high antifungal properties (Liu et al., 2017). Antimicrobial activity of TiO_2_ or in binary compositions carried out in medicine or food science. The TiO_2_/ZnO supported in 4A zeolite showed superior activity as antimicrobial agent (Azizi‐Lalabadi et al., 2019). Ansari et al. (2020) showed that TiO_2_ nanofibers were more active against Gram‐negative *Pseudomonas aeruginosa* cells than Gram‐positive *Staphylococcus aureus*. Also, the antibacterial and antibiofilm results suggested that TiO_2_ can be utilized for coating different inanimate objects, in food packaging and in wastewater treatment, and purification for preventing bacterial growth. In other research, the Fe‐doped TiO_2_/bamboo exhibited a much higher inhibition ability to mold fungi compared with original bamboo and TiO_2_/bamboo, under the natural environment (Li et al., 2017). Based on Kim et al. (2019) results, thorn‐like TiO_2_ nanoarrays physically punctured the cell membrane of bacteria.

As far as we know, there is no report about the antifungal activity of TiO_2_/AgBr nanocomposites. Given the above discussions, we prepared binary TiO_2_/AgBr nanocomposites by a simple ultrasonic‐aided method. Then, these nanocomposites were studied by different instruments such as FT‐IR, SEM, EDX, and XRD. The antifungal activity of TiO_2_/AgBr nanocomposites was evaluated against *F. graminearum*, *B. cinerea*, and *S. sclerotiorum*. The results exhibited that the TiO_2_/AgBr (20%) sample acts as a highly effective nanocomposite for antifungal property compared to the other samples.

## 2 MATERIALS AND METHODS

### 2.1 Instruments

The phase structure of the materials was studied by Philips Xpert XRD, applying CuKα radiation. The morphological features and chemical composition of the samples were characterized by LEO 1430 VP SEM/EDX instrument. The FT‐IR spectra were provided by a PerkinElmer Spectrum RX I instrument. The ultrasonic treatment was applied with a Bandelin ultrasound generator HD 3100.

### 2.2 Preparation of the nanocomposites

**FIGURE 1:**
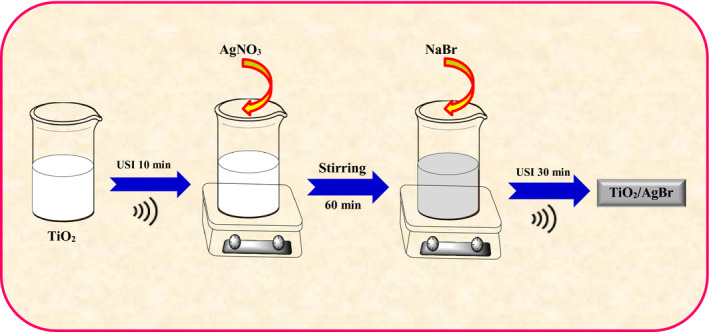
Schematic image for the synthesis of TiO_2_/AgBr samples

All chemicals had high purity, and deionized water was used during this study. The TiO_2_/AgBr nanocomposites, with the AgBr content of 20 wt%, were synthesized as follows: 0.4 g TiO_2_ (P25) was first sonicated in water (150 ml) with ultrasonication for 10 min. Then, 0.09 g AgNO_3_ (Loba Chemie) was added into the solution with stirring. Next, afterward, a 20 ml aqueous solution containing 0.054 g NaBr (Loba Chemie) was drop‐wisely appended into the solution and followed for 1 hr vigorous stirring and the suspension was sonicated for 60 min. Finally, the produced precipitate was filtered and washed two times with water, and then air‐dried at 60°C (Figure 1).

### 2.3 The utilized fungi

Two phytopathogenic fungal strains *B. cinerea* (FCUM672), and *S. sclerotiorum* (FCUM373) provided by the Fungal Collection of University of Mohaghegh Ardabili. *Fusarium graminearum* (CBS130604) was obtained from the CBS Culture Collection (CBS‐KNAW Westerdijk Fungal Biodiversity Centre, Utrecht, Netherland). All strains were cultured on PDA (potato dextrose agar, Merck) medium and were preserved on SNA (slant synthetic nutrient‐poor agar) at 4°C.

### 2.4 Effect of nanocomposites on the fungi spores in the bioreactor

The growth of *F. graminearum* spores was realized on the SNA at 25°C for 7 days. After washing the plates with sterile water and separating spores by mechanical agitation, the spore suspension was set on 1 × 10^4^ spores/ml via a hemacytometer. The antifungal effect of the nanocomposite on the *F. graminearum* spores was carried out according to the related method (Sichel et al., 2007). A wooden enclosure and a two‐walled Pyrex reactor were attached to a thermostat and the solution temperature was kept at 25°C. To avoid the adverse light effects, the experiments were performed in the dark condition. The fungal spore suspension and 0.002 g of the nanocomposite were transferred to one reactor, and the fungal spore suspension alone as control was added to the other reactor. At different time intervals, the sampling from each reactor was performed using a micro sampler. Samples were inoculated into pellets containing MEA (Malt Extract Agar) medium and incubated in the dark medium for 15 hr. Germinated spores were counted and compared with the control using a stereo microscope.

### 2.5 Effects of nanocomposite on fungi mycelial growth

The nanocomposite was mixed with PDA medium according to the method known as the microdilution (Kaur et al., 2012). A 5‐mm mycelial disk from fresh cultures of target fungi was placed in the middle of with PDA medium containing 100, 150, 200, 300, and 400 ppm of the nanocomposite. The inoculated and the control (PDA medium without the nanocomposite) Petri dishes were incubated at 25°C for 16 hr light and 8 hr dark. The growth of the mycelial colony in each fungus was measured daily until filled by the fungi grown in the control plates. It was elongated 4, 5, and 7 days for *S. sclerotiorum*, *B. cinerea*, and *F. graminearum*, respectively. Three replicates were considered for each treatment, and mycelial growth inhibition percentages of different concentrations were calculated using the following formula:

Inhibition Percentage(%)=(R‐r)/R,

where *R* and *r* are the diameter of the fungus colony in control and treated plates, respectively. Analysis of variance and probit analysis of inhibition percentage were performed using SPSS software version 24. Also, the observed rate constant (*k*_obs_) of the inactivation processes over the materials were obtained by the slope of ln (*N_t_*/*N_o_*) = −*kt*, in which *N_o_* and *N_t_* are the initial and at time of *t* fungus population (in cfu/ml), respectively.

## 3 RESULTS

**FIGURE 2:**
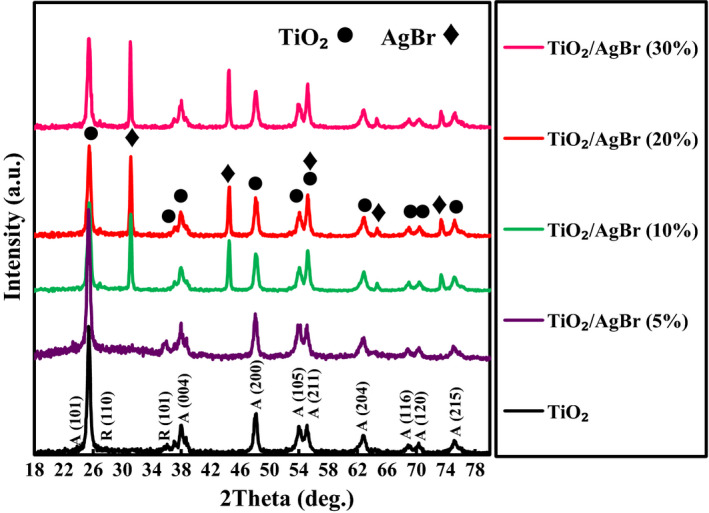
XRD patterns for the fabricated materials

To investigate the phase structure and purity of the materials, the XRD tests were used. The XRD patterns of the as‐fabricated samples are shown in Figure 2. For the TiO_2_, the diffraction peaks belong to the tetragonal phase (JCPDS no. 04‐0477) (Feizpoor et al., 2019). For the TiO_2_/AgBr nanocomposites, in addition to the peaks belonged to TiO_2_, characteristic peaks of AgBr were also observed (JCPDS no. 79‐0149) (Pirhashemi & Habibi‐Yangjeh, 2016). Because of the small amount of AgBr in the TiO_2_/AgBr (5%) sample, no characteristic peaks for AgBr were detected in the TiO_2_/AgBr (5%) sample. These patterns show the successful combining of TiO_2_ and AgBr to build the TiO_2_/AgBr nanocomposites.

**FIGURE 3:**
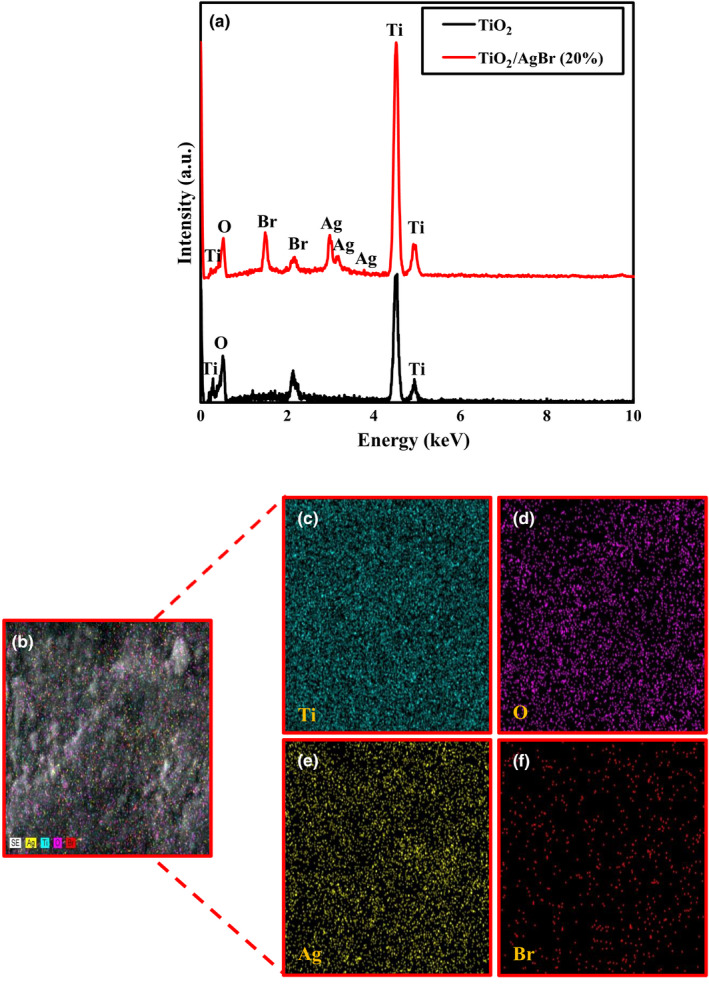
EDX results for the TiO_2_ and TiO_2_/AgBr (20%) samples (a), and EDX mapping of the TiO_2_/AgBr (20%) nanocomposite (b–f)

To evaluate the presence of expected elements in the samples, EDX analyses were employed. The corresponding EDX spectra of TiO_2_ and TiO_2_/AgBr (20%) samples are provided in Figure 3a, indicating Ti and O elements in the TiO_2_ sample without any impurities, whereas the TiO_2_/AgBr (20%) sample consists of Ti, O, Br, and Ag elements. The above results indicated that AgBr grows on the surface of the TiO_2_ sample. EDX mapping was obtained to investigate further the distribution of the elements in the TiO_2_/AgBr (20%) sample (Figure 3(b–f)). Based on the results, Ti, O, Br, and Ag elements are realized distributed in the sample, confirming that the TiO_2_/AgBr (20%) sample has been successfully synthesized.

**FIGURE 4:**
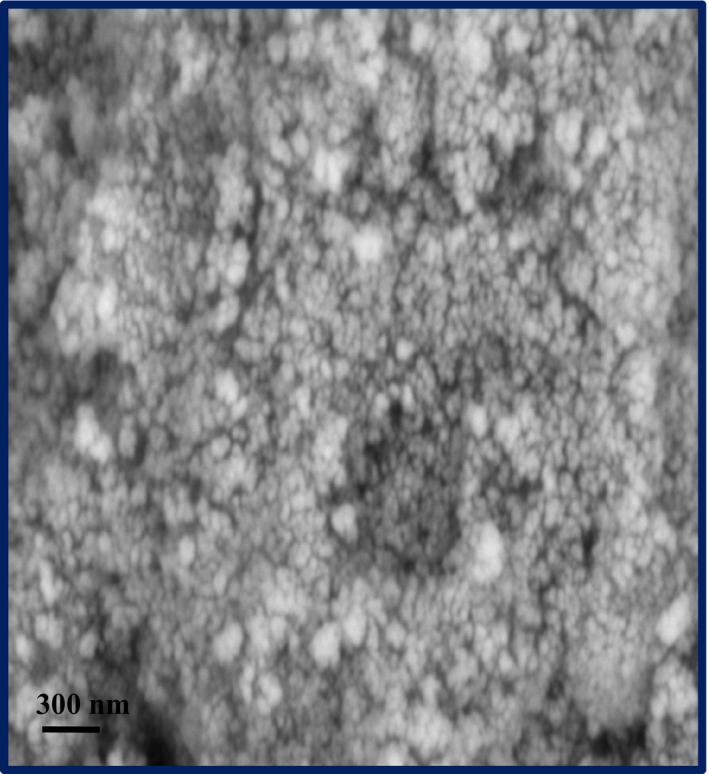
SEM image of the TiO_2_/AgBr (20%) sample

The structure and morphology of the TiO_2_/AgBr (20%) sample were inspected using SEM analysis. Figure 4 reveals the corresponding SEM image of the TiO_2_/AgBr (20%) sample. From this figure, the TiO_2_/AgBr (20%) nanocomposite presents spherical morphology with high aggregation.

**FIGURE 5:**
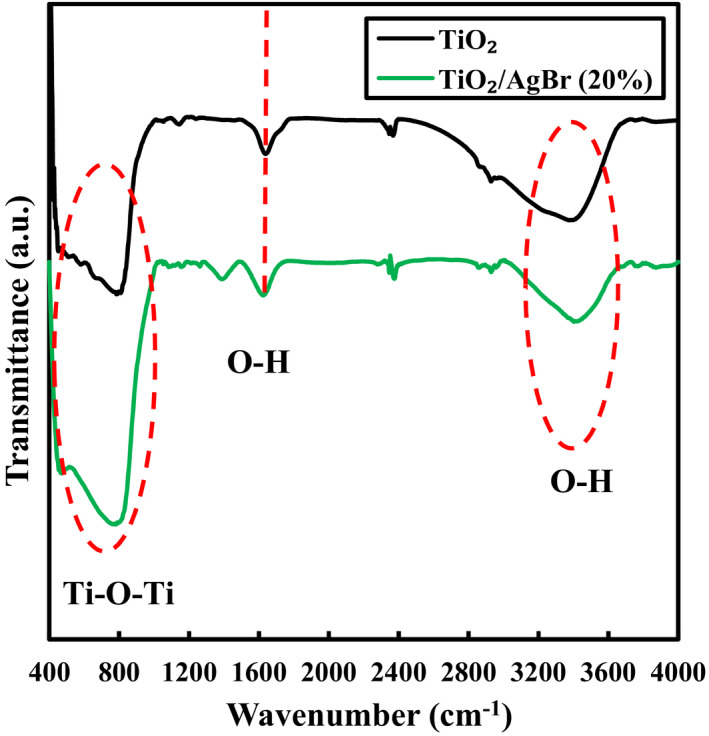
FT‐IR spectra for the TiO_2_ and TiO_2_/AgBr (20%) samples

To investigate the functional structure of the samples, FT‐IR technique was performed. As shown in Figure 5, all materials show absorption bands at 3400–3600 cm^−1^ and in the 400–700 cm^−1^ area, which are assigned to the O–H and Ti–O bonds, as the vibrational stretching modes (Fang et al., 2017; Zhu et al., 2015). Finally, like to the other articles about silver halides in the FT‐IR spectra, the peaks for the Ag–Br bond in 400–4000 cm^−1^ are not observed (Pirhashemi & Habibi‐Yangjeh, 2016).

**FIGURE 6:**
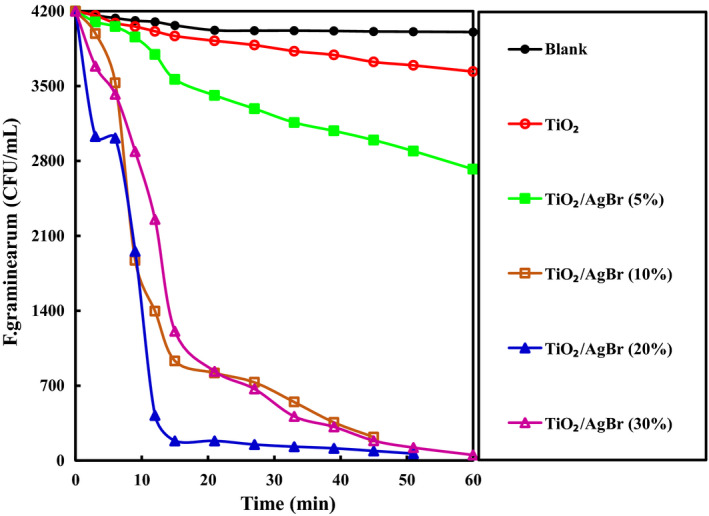
Inactivation of *Fusarium graminearum* over the TiO_2_ and TiO_2_/AgBr nanocomposites

The antifungal activity of as‐obtained samples was studied by the inactivation of *F. graminearum* spores, as are shown in Figure 6. It is evident that under the provided conditions, no considerable inactivation of *F. graminearum* was taken in the absence of nanocomposite, indicating the fungus possesses high stability. Interestingly, the TiO_2_/AgBr samples exhibited enhanced activity than the bare TiO_2_. By increasing the weight percentage of AgBr to 20%, the antifungal activity of binary samples was quickly enhanced, and then reduced with adding further AgBr. Inactivation of fungal spores after 60 min was 35.2%, 97.8%, 98.9%, and 98.7%, respectively, for the 5%, 10%, 20%, and 30% of AgBr, while the inhibition rate was 13.4% for the pure TiO_2_. Thus, the outcomes demonstrated that the 20% TiO_2_/AgBr nanocomposite exhibited the best antifungal performance.

**FIGURE 7:**
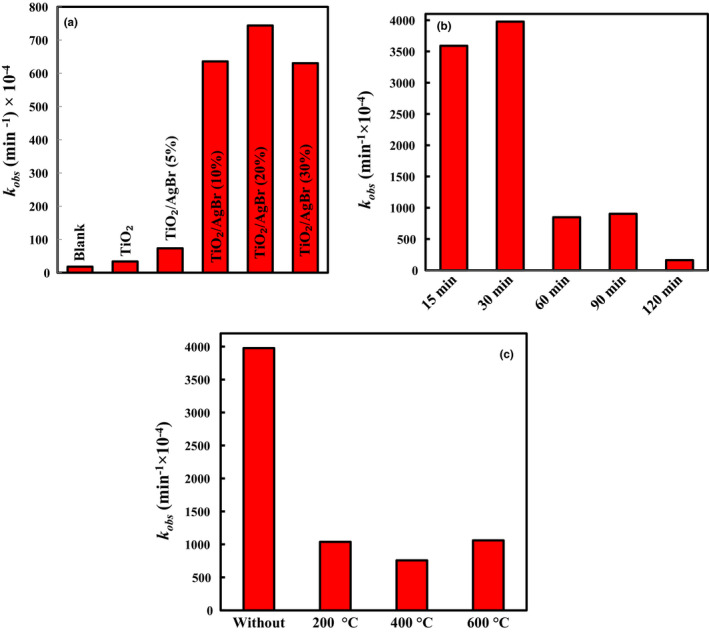
The inactivation rate constants of *Fusarium graminearum*: (a) over the materials, (b) over the TiO_2_ and TiO_2_/AgBr (20%) nanocomposite prepared at different ultrasonic‐irradiation times, (c) over the TiO_2_/AgBr (20%) nanocomposite calcined at different temperatures

Inactivation rate constants of *F. graminearum* obey the pseudo‐first‐order kinetics model, as displayed in Figure 7a. The rate constant over the TiO_2_ sample is 33.6 × 10^–4^ min^−1^. The 20% TiO_2_/AgBr sample displayed the highest rate of constant (744 × 10^–4^ min^−1^), which is 22.1 times higher than the TiO_2_ sample. The order of the antifungal activity is TiO_2_ < TiO_2_/AgBr (5%) < TiO_2_/AgBr (30%) < TiO_2_/AgBr (10%) < TiO_2_/AgBr (20%). The results demonstrate that the integration of TiO_2_ with AgBr can greatly enhance the antifungal property.

The preparation time of samples could primarily affect their crystallinity and size of the particles. Thus, the 20% TiO_2_/AgBr sample was fabricated by ultrasonic irradiations for 15, 30, 60, 90, and 120 min. As can be seen in Figure 7b, the antifungal activity reduces with increased preparation time, and the sample fabricated by ultrasonic irradiation for 30 min possesses the highest antifungal activity.

The influence of calcination temperature on the antifungal activity of the 20% TiO_2_/AgBr sample was studied, and the results are shown in Figure 7c. The sample fabricated by ultrasonic irradiation for 30 min was calcined for 120 min at 200, 400, and 600°C. It can be seen that the inactivation rate constant of the noncalcined nanocomposite is much higher than that of the calcined samples.

**FIGURE 8:**
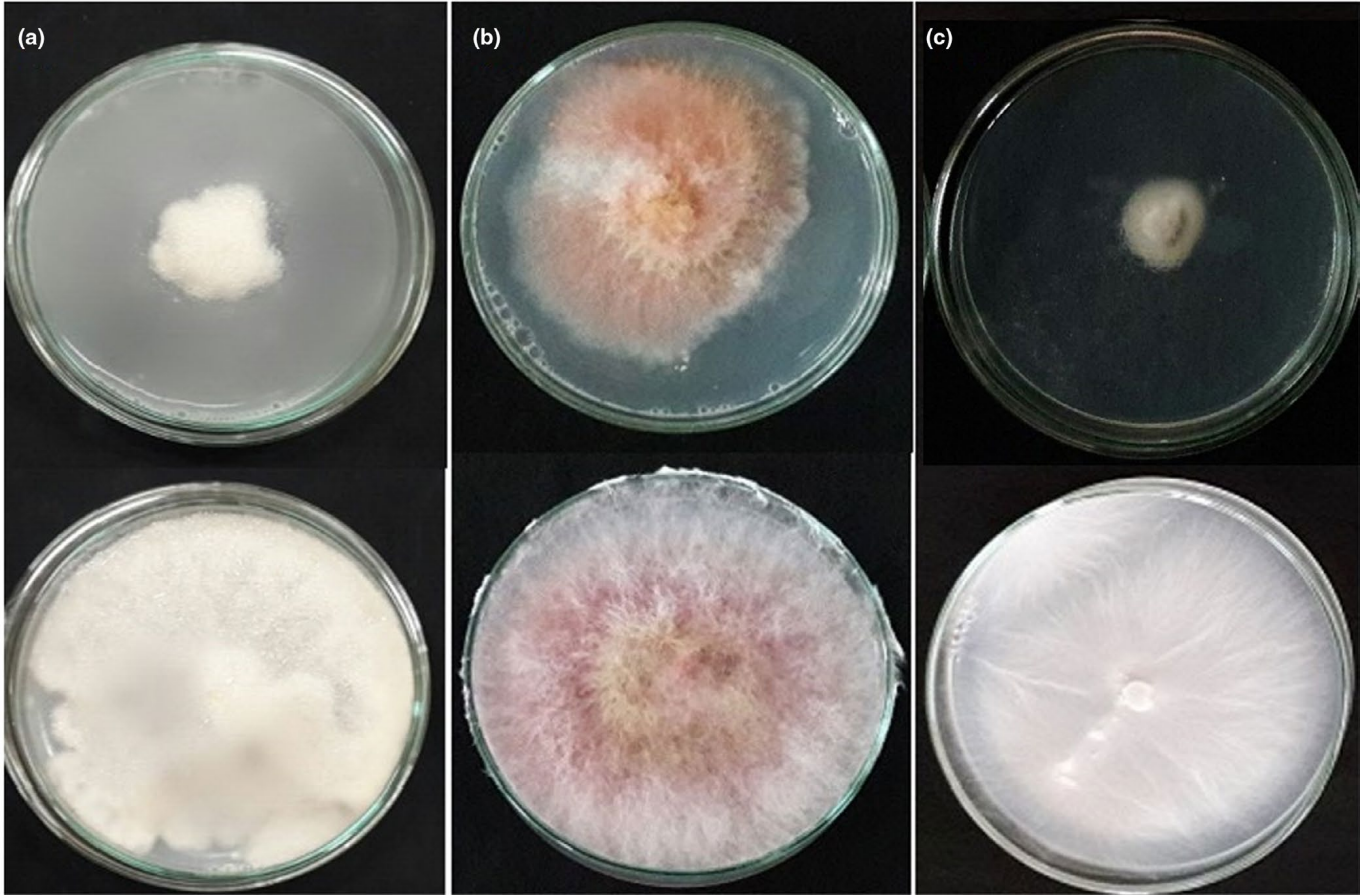
Inhibition effect of TiO_2_/AgBr (20%) on the mycelial growth of phytopathogenic fungi: (a) *Botrytis cinerea*, (b) *Fusarium graminearum*, and (c) *Sclerotinia sclerotiorum*. Above row: treatment with 400 ppm of the nanocomposite and below row: control

The TiO_2_/AgBr nanocomposites also has considerable inhibition on the mycelial growth of phytopathogenic fungi (Figure 8). Analysis of variance revealed that different concentrations of the nanocomposite (*F* = 142.051, *df* = 3, 24, *p* < .0001 for *S. sclerotiorum*; *F* = 17.727, *df* = 3, 32, *p* < .0001 for *F. graminearum*; and *F* = 36.548, *df* = 3, 24, *p* < .0001 for *B. cinerea*) and exposure times (*F* = 23.559, *df* = 2, 24, *p* < .0001 for *S. sclerotiorum*; *F* = 21.229, *df* = 3, 32, *p* < .0001 for *F. graminearum;* and *F* = 17.797, *df* = 2, 24, *p* < .0001 for *B. cinerea*) significantly inhibited the mycelial growth of fungi. The interaction of nanocomposite concentrations and exposure times was also significant on the mycelial growth inhibition of *S. sclerotiorum* (*F* = 6.320, *df* = 6, 24, *p* = .0004) and *B. cinerea* (*F* = 7.280, *df* = 6, 24, *p* = .0001), but it was not significant in the case of *F. graminearum* (*F* = 0.881, *df* = 9, 32, *p* = .5514).

The results of probit analysis for data obtained from the TiO_2_/AgBr nanocomposites on the mycelial growth inhibition of phytopathogenic fungi at different exposure times are shown in Table 1. The concentration needed for 50% mycelial growth inhibition (IP_50_ value) of *F. graminearum* was 429.94 and 143.80 ppm after 4 and 7 days, respectively. On the other hand, the IP_50_ values were significantly decreased according to the increase of exposure time. The same condition can be found for *B. cinerea*, while in the case of *S. sclerotiorum*, the IP_50_ values were enhanced as the exposure time increased. Furthermore, it can be seen that *S. sclerotiorum* with its low IP_50_ (319.992 ppm) was more susceptible than *B. cinerea* after 4 days.

**TABLE 1:** Probit analysis of TiO_2_/AgBr nanocomposite on the mycelial growth inhibition of *Fusarium graminearum*, *Botrytis cinerea*, and *Sclerotinia sclerotiorum* at different exposure times

Fungi	Time (day)	Inhibition Percentage 50%			
		(90% Fiducial Limit) (ppm)	Chi‐square (*df* = 2)	Slop	Significant
*F. graminearum*	2	901.81 (nc)	6.01	0.81	0.058^a^
	4	429.94 (312.91–974.75)	0.9	1.07	0.642^a^
	5	324.19 (254.62–570.68)	2.17	1.05	0.351^a^
	7	143.80 (41.034–208.88)	0.35	0.77	0.844^a^
*B. cinerea*	2	2,419.26 (nc)	0.39	0.53	0.823^a^
	4	522.12 (nc)	40.42	4.16	0.001^b^
	5	400.00 (387.61–416.43)	0.001	16.67	1.007^a^
*S. sclerotiorum*	2	229.536 (nc)	6.281	1.94	0.044^b^
	3	301.064 (nc)	7.029	2.555	0.030^b^
	4	319.992 (298.788–346.048)	2.942	5.091	0.230^a^

Note

nc is a noun calculated.

^a^ Because the significant level is greater than 0.05, no heterogeneity factor is used in the calculation of the fiducial limit.

^b^ Because the significant level is less than 0.05, a heterogeneity factor is used in the calculation of the fiducial limit.

## 4 DISCUSSION

Briefly, binary TiO_2_/AgBr nanocomposites were synthesized using a facile ultrasonic irradiation route, and they were characterized by various instruments. After adding AgBr nanoparticles to the surface of TiO_2_, the antifungal activity was markedly enhanced. This boosted antifungal activity in the binary nanocomposite was ascribed to the synergistic interactions between TiO_2_ and AgBr. Silver bromide, as an Ag‐based material, possesses antifungal activity. Silver ions in AgBr have a broad antimicrobial spectrum and can inhibit the growth of fungi (Zhang et al., 2017). The weight percentage of silver bromide affects the antifungal activity of binary nanocomposites. The sample with 20 wt% of silver bromide represented the highest inhibitory on the mycelial growth of *F. graminearum*.

Furthermore, the inactivation rate decreased with increasing ultrasound irradiation time. Reducing the antifungal properties of the nanocomposite by increasing the preparation time can be ascribed to the aggregation of nanoparticles (Hoseinzadeh et al., 2016). It was also found that the antifungal activity of the nanocomposite without calcination was higher than those of the calcined samples. It is in accordance with the report of Hoseinzadeh et al. (2016) which attributed to the reduction of the nanocomposite surface area at high temperatures due to particle agglomeration and the size growth (Hoseinzadeh et al., 2016; Singh et al., 2014). The TiO_2_/AgBr nanocomposites also have significant inhibition on the survival of fungi mycelium. The nanocomposite concentration and exposure time were also significant effects on the mycelial growth inhibition of *S. sclerotiorum* and *B. cinerea*. The results illustrated that the TiO_2_/AgBr (20%) sample possesses notably higher antifungal abilities than the other related works. For example, Hoseinzadeh et al. (2016) prepared Fe_3_O_4_/ZnO/AgBr nanocomposites with a microwave‐assisted approach and displayed an inactivation rate constant 395 × 10^–4^ min^−1^ against *F. graminearum* over the Fe_3_O_4_/ZnO/AgBr (1:8) under visible‐light irradiation. The antifungal activity of the present nanocomposite is 9.8‐folds higher than the Fe_3_O_4_/ZnO/AgBr (1:8) sample in the inactivation of *F. graminearum*. According to the notable antifungal activity, simple synthesis, and eco‐friendly nature, TiO2/AgBr nanocomposites can be recommended as safe and sound alternatives to synthetic chemicals in the management of the plant‐pathogenic fungi. Also, the investigation of antifungal mechanism of TiO2/AgBr nanocomposites is suggested for further studies.

## CONFLICT OF INTEREST

The authors do not have any conflict of interest to declare.

## AUTHOR CONTRIBUTIONS

**Aziz Habibi‐Yangjeh:** Conceptualization (lead); Data curation (lead); Formal analysis (equal); Funding acquisition (lead); Investigation (lead); Methodology (lead); Project administration (lead); Resources (lead); Software (equal); Supervision (lead); Validation (equal); Visualization (equal); Writing‐original draft (equal); Writing‐review & editing (lead). **Mahdi Davari:** Conceptualization (equal); Data curation (equal); Formal analysis (equal); Funding acquisition (equal); Investigation (equal); Methodology (equal); Project administration (equal); Resources (equal); Software (supporting); Supervision (equal); Validation (equal); Visualization (equal); Writing‐original draft (equal); Writing‐review & editing (equal). **Reza Manafi‐Yeldagermani:** Conceptualization (equal); Formal analysis (equal); Investigation (equal); Methodology (equal). **Shervin Alikhah Asl:** Conceptualization (equal); Formal analysis (equal); Investigation (equal); Methodology (equal). **Samira Enaiati:** Conceptualization (equal); Formal analysis (equal); Investigation (equal); Methodology (equal); Software (equal). **Asgar Ebadollahi:** Formal analysis (equal); Methodology (equal); Resources (equal); Validation (equal); Writing‐original draft (equal); Writing‐review & editing (equal). **Solmaz Feizpoor:** Formal analysis (equal); Methodology (equal); Resources (equal); Validation (equal); Writing‐original draft (equal); Writing‐review & editing (equal).

## ETHICAL APPROVAL

This study does not involve any human or animal testing.

## Data Availability

The data that support the findings of this study are available from the corresponding author upon reasonable request.
